# Quantitative evaluation of age-related decline in control of preprogramed movement

**DOI:** 10.1371/journal.pone.0188657

**Published:** 2017-11-29

**Authors:** Naoshi Shimoda, Jongho Lee, Mitsuhiko Kodama, Shinji Kakei, Yoshihisa Masakado

**Affiliations:** 1 Department of Rehabilitation Medicine, Tokai University School of Medicine, Kanagawa, Japan; 2 Movement Disorders Project, Tokyo Metropolitan Institute of Medical Science, Tokyo, Japan; Nanjing University, CHINA

## Abstract

In this paper, we examined the age-related changes in control of preprogramed movement, with emphasis on its accuracy. Forty-nine healthy subjects participated in this study, and were divided into three groups depending on their ages: the young group (20–39 years) (n = 16), the middle-age group (40–59 years) (n = 16), and the elderly group (60–79 years) (n = 17). We asked the subjects to perform step-tracking movements of the wrist joint with a manipulandum, and recorded the movements. We evaluated the accuracy of control of preprogramed movement in the three groups in terms of the primary submovement, which was identified as the first segment of the step-tracking movement based on the bell-shaped velocity profile, and calculated the distance between the end position of the primary submovement and the target (i.e. error). The error in the young group was found to be significantly smaller than that in the middle-age and elderly groups, i.e., the error was larger for the higher age groups. These results suggest that young subjects have better control of preprogramed movement than middle-age or elderly subjects. Finally, we examined the temporal property of the primary submovement and its age-related changes. The duration of the primary submovement tended to be longer for the aged groups, although significance was reached only for the elderly group. In particular, the ratio of the duration of the primary submovement to total movement time tended to be lower for the aged groups, suggesting that the proportion of additional movements that are required to compensate for the incomplete control in the preprogramed movement, which are under feedback control, was higher for the aged groups. Consequently, our results indicate that the distance between the end point of the primary submovement and the target center (i.e. error) in the step-tracking movement is a useful parameter to evaluate the age-related changes in control of preprogramed movement.

## Introduction

The motor skills of elderly persons are known to gradually deteriorate with age, with a decrease in the velocity and accuracy of their movements. In the field of neurorehabilitation, improvement in the evaluation methods of hand function and the objective and quantitative collection of data is very important, since they may help provide a standard definition of clinically important improvement as part of the neurorehabilitation strategy in patients with neuromuscular disorders or stroke. In particular, the functions that deteriorate with aging require careful evaluation.

Several previous studies quantified motor function in elderly persons by studying their arm movements [1–3]. For instance, Ketcham et al. [2] reported the kinematic characteristics of elderly subjects, i.e. the slowness of their movements compared with young subjects, by using experimental tasks with variable levels of difficulty. The level of difficulty of the task was altered by varying the size of the movement target and the kinematic properties of the arm movements. However, in order to properly evaluate age-related motor ability, it is necessary to more precisely analyze movement accuracy, assessing motor control as well as extracting the characteristics of the movement kinematics, rather than evaluating maximum movement velocity alone [4].

It is generally accepted that motor control by the central nervous system consists of feedback (FB) control and feedforward (FF) control. FF control is based on experiences, while FB control is based on visual information and/or other sensory modalities [5,6]. When subjects make a rapid, goal-directed movement, it is generally constructed of two components: the primary and secondary submovements [7]. The primary submovement is characterized by a roughly straight trajectory and a bell-shaped tangential velocity profile [8–12]. It is most likely that FF control plays the primary role in initiation of the primary submovement. Unfortunately, FF control is not always perfect, and subjects sometimes miss the target. Therefore, they need to generate an additional movement near the end of the movement (i.e. the secondary submovement) with FB control [5,8,9,13] when there remains an error in performance of the task.

In order to analyze the age-related changes in the two components of motor control, previous studies employed a step-tracking movement or a rapid point-to-point reaching movement [2,3,7,14–22]. Some studies reported significant age-related changes in the precision of motor control [2,14,16,18–20], while others [3,17,19,21] reported that the changes were insignificant. Thus, there is no consensus regarding the age-related differences in the accuracy of movements. The purpose of this study was to examine age-related changes in the precision of control of preprogramed movement for a rapid step-tracking movement of the wrist joint. More specifically, in order to analyze the age-related changes in control of preprogramed movement, we focused on the parameters of the primary submovement that are characterized by a bell-shaped velocity profile in the first part of the step-tracking movement. To evaluate the accuracy of control of preprogramed movement, we calculated the distance between the end point of the primary submovement and the target center (i.e. error), and examined age-related changes in the error. We also examined age-related changes in the duration of the primary submovement in different age groups. We found these parameters useful to evaluate age-related changes in control of preprogramed movement.

## Materials and methods

### Subjects

The present study included three groups of subjects. The first group consisted of 16 young healthy adults (young group: 20–39 years; mean age, 29.9 ± 6.8 years; 9 men and 7 women). The second group included 16 healthy middle-aged adults (middle-aged group: 40–59 years; mean age, 49.3 ± 6.2 years; 6 men and 10 women). The third group consisted of 17 healthy elderly adults (elderly group: 60–79 years; mean age, 68.6 ± 4.8 years; 11 men and 6 women). All subjects were right-handed according to the Edinburgh handedness scale [23]. They reported no history of neurological illness or psychiatric history and were not taking regular medication. The present study was approved by the Clinical Research Review Committee of the Tokai University School of Medicine, and was performed in accordance with the Declaration of Helsinki. Informed consent was obtained in writing from all subjects prior to their participation in the experiments.

### Experimental setup

An outline of the experimental system is shown in S1 Fig. It consisted of four elements: a wrist joint manipulandum, a notebook computer for data recording and analysis, a small Universal Serial Bus (USB) analog-to-digital (A/D) converter interface, and a measurement device [24]. Each subject sat in a chair, rested his or her right forearm on an armrest, and grasped a Strick-Hoffman type manipulandum ([25–27], Hoyo Elemec Co., Ltd., Sendai, Japan) with his or her right hand. The manipulandum could be freely rotated around the horizontal and vertical axes. The wrist movements were performed with two degrees of freedom (extension-flexion, radial-ulnar flexion) and were measured using two position sensors on the manipulandum. The movements were reflected by a cursor on a computer screen (black dot 2 mm in diameter). In other words, the center position of the manipulandum corresponded to the center of the screen, and the cursor moved left for flexion movements, right for extension movements, up for radial deviation, and down for ulnar deviations of the wrist. The target was displayed as a circle, and the diameter of the target (1 cm) corresponded to a wrist movement of 4.5°. The cursor on the PC screen was linked with the movement of the subject’s wrist during each experiment. To perform the wrist movement task, the subjects maintained their right forearm in the neutral position, midway between full pronation and full supination (see Fig 1 in [28]).

### Movement task

**Fig 1:**
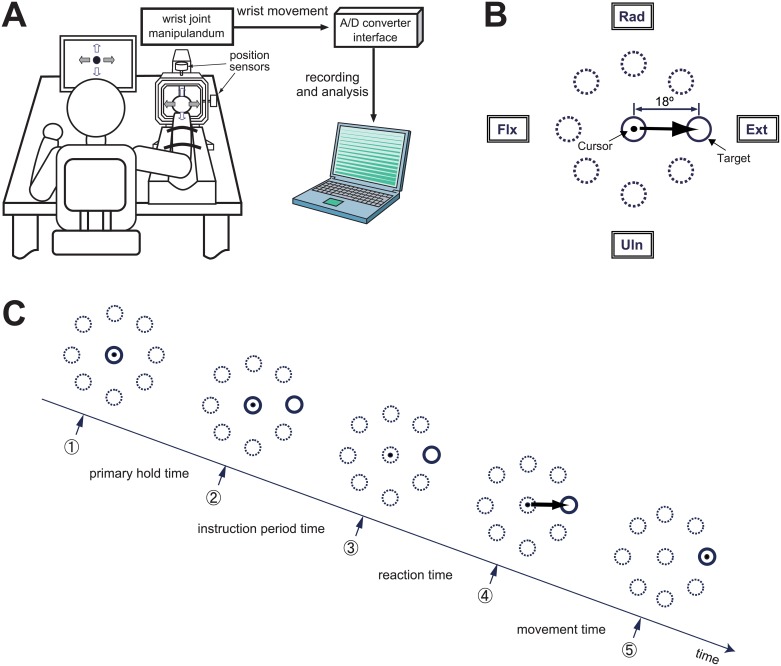
Experimental design and movement task. (A) Experimental setup. Each subject sat approximately 60 cm in front of a computer screen that displayed a cursor and a target, and grasped a Strick-Hoffman type manipulandum with his/her right hand [30]. The handle of the manipulandum rotated freely around the horizontal and vertical axes with low-friction. To perform the wrist movement task, the subject maintained his/her forearm in the neutral position, midway between full pronation and full supination. Two position sensors that were coupled to the device measured the angle of the wrist in the planes of flexion-extension and radial-ulnar deviation. (B) Presentation of the targets and required movement of a cursor for the step-tracking task used in this study. The target was displayed as an open circle with an internal diameter that corresponded to a wrist movement of 4.5°. The cursor was a black point (approximately 2 mm in diameter) that moved in proportion to the subject's wrist movements. The position of the cursor in the center target corresponded to the center of the screen, and the cursor moved left for flexion movements, right for extension movements, up for radial deviations, and down for ulnar deviations of the wrist. The subjects were required to move the cursor from the center target to one of eight targets that were randomly presented, as rapidly and accurately as possible. (C) Procedure of the step-tracking task. First, the subject put the cursor in the central target. After 500 msec (*primary hold time*), a new target was randomly displayed in one of eight directions. After 500–1000 ms (*instruction period time*), the central target disappeared [start signal, (3) in figure], at which time the subject had to start moving the cursor. The subject was supposed to put the cursor in the new target as precisely and fast as possible.

The step-tracking task performed in the present study is shown in Fig 1B. The task began with the cursor positioned in a circular target displayed in the middle of the computer screen. A new target appeared randomly in one of eight directions around the central circle. The subject was required to move the cursor as quickly and accurately as possible to the new target with a single step of wrist movements. The procedure of the step-tracking task is shown in Fig 1C. After a hold period of 500 ms (*primary hold time* in Fig 1C), a new target was displayed at a location requiring an 18° movement of the wrist joint to reach it from the central target ((2) in Fig 1C). The central target disappeared after a variable period (500–1000 ms, *instruction period time* in Fig 1C), and the subject was required to move the cursor immediately to the new target as rapidly and accurately as possible with a one-step movement ((3)-(5) in Fig 1C). About 2 s after the subject moved the cursor onto the specified target, the central target was displayed again. The subject returned the cursor to the center target and waited for the next new target to be displayed. In this task, one complete set of movements was defined by one movement from the central target to each of the eight peripheral targets. After performing one practice set (eight attempts), each subject performed eight such sets (total of 64 trials). The eight targets in each set were displayed in random order.

### Data analysis and analysis parameters

When performing the experimental task, the position of the wrist (X, Y) was recorded at a sampling rate of 1 kHz. Signals from the potentiometers were filtered by a Butterworth third-order low-pass filter (cut-off frequency, 8Hz) and recorded as the wrist position (X and Y). Filtered signals were differentiated to derive values for velocity. In this study, we only analyzed wrist movements to the right (extension) and left (flexion) targets from among the eight targets, and analyzed the characteristics of motor function and control for our three groups of subjects. The age-related changes in motor control ability were assessed using three parameters: reaction time (RT), movement time (MT), maximum velocity (MVel), and terminal error. These were defined as follows:

#### 1) Reaction time (RT)

RT was defined as the time period from display of the central target (start sign) to the onset of wrist movement (see *reaction time* in Fig 1C).

#### 2) Movement time (MT)

MT was defined as the period from the onset of cursor movement to the time when the cursor entered the target and stopped moving.

#### 3) Maximum velocity (MVel)

MVel was defined as the maximum value of tangential velocity during movements.

#### 4) Terminal error

Terminal error was defined as the error between the final end-point and the target.

**Fig 2:**
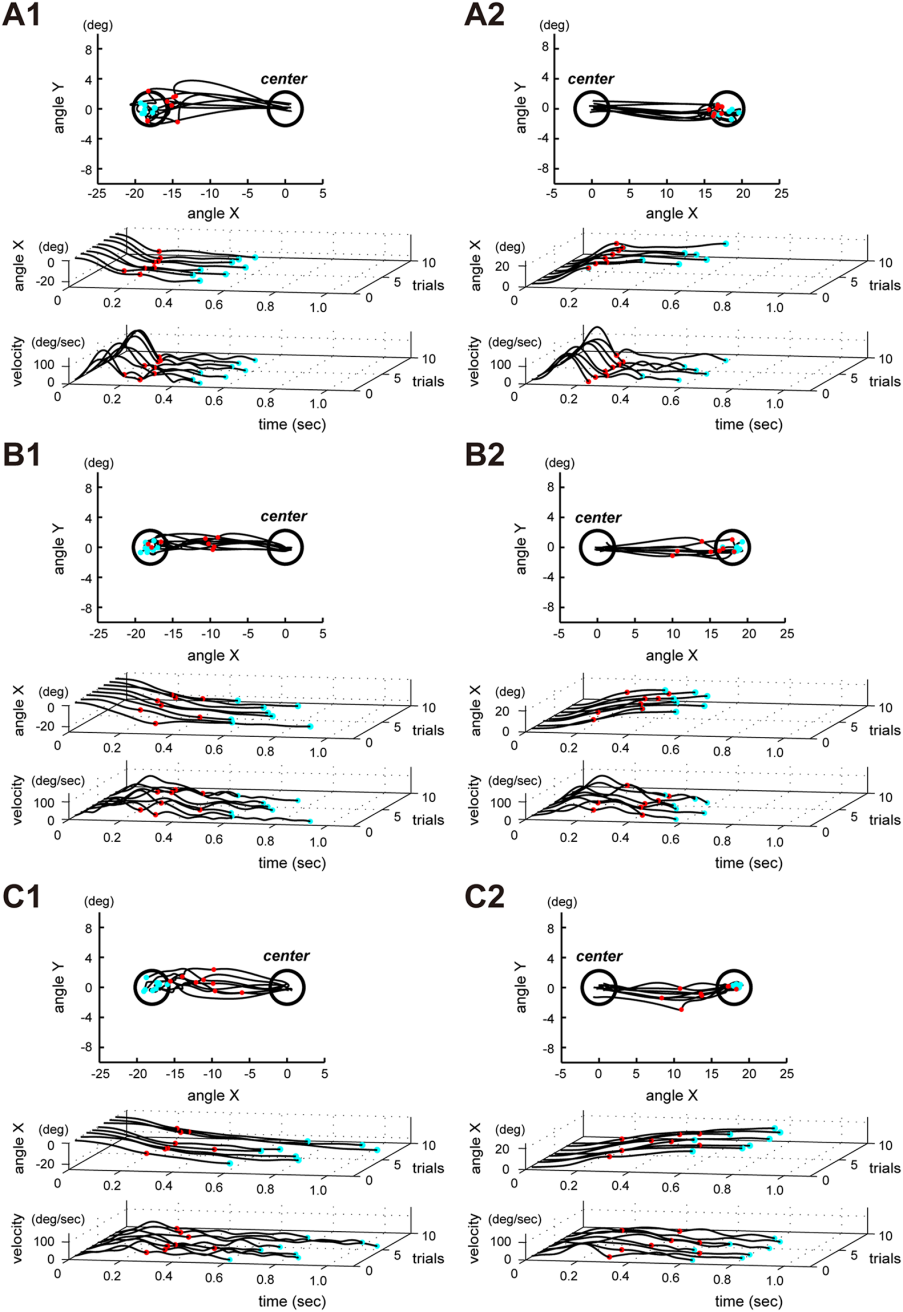
A typical example of step-tracking movements in the three age groups. (A1) An example of the wrist flexion movement in young subjects. The black lines in the inset represent the movement trajectories, while those in the top and bottom traces show the corresponding X-axis component of wrist joint angle (shortly, angle X) and the corresponding tangential velocity profiles, respectively. Also, the red circles on the trajectories, the angle X and the velocity profiles show the end of the primary submovements, while the cyan points represent the final end-points for the step-tracking movements. (A2) An example of the wrist extension movement in young subjects. (B1) An example of the wrist flexion movement in middle-age subjects. (B2) An example of the wrist extension movement in middle-age subjects. (C1) An example of the wrist flexion movement in elderly subjects. (C2) An example of the wrist extension movement in elderly subjects. Note prolongation of the time required to arrive at the target, and the wider bell-shaped velocity profile for the aged groups. In particular, the angle X of the 20s-30s group is the shape of critical damping (ζ = 1) (ζ: damping ratio), while the angle X for the 60s-70s group took the form of an "overdamped feedback system (ζ> 1) [31]. Also, the red circles (end of primary submovement) were situated further away from the target in elderly subjects, and the duration of movement was longer than in younger subjects.

We also examined the age-related differences in the ability of motor control in this study. In particular, we extracted a parameter characterizing the accuracy of control of preprogramed movement. For this, we analyzed the primary submovement in the step-tracking movement, examining the ability of control of preprogramed movement and its age-related changes. We first identified the primary submovement by extracting the end of the initial bell-shaped velocity profile, where the first minimum velocity value was attained after the maximum velocity, and represented the end of the primary submovement as a red circle in Fig 2. Then, we calculated the following two parameters: the distance between the end of the primary submovement and the center of the target (i.e. error) and the duration of the primary submovement. These parameters were used to analyze differences in control ability of preprogramed movement between the three age groups.

#### 5) Error of primary submovement

The error of the primary submovement was defined as the distance between the end of the primary submovement and the center of the target. The end points of primary submovements (red circles in Fig 2) were identified by extracting the first point from the negative to positive value on the velocity profile, based on zero-crossing positions in the acceleration profile [7].

#### 6) Duration of primary submovement

The duration of the primary submovement was calculated as the time from the onset of the movement to the end point of the primary submovement.

### Statistical tests

Group differences were assessed by nonparametric analysis using the Kruskal-Wallis test (*Kruskal-Wallis* function in the statistics toolbox of Matlab, Ver. 7.11.0.584 (R2010b), Mathworks, MA) for three groups, and the Mann-Whitney U test (*Ranksum* function in the statistics toolbox of Matlab, Ver. 7.11.0.584(R2010b), Mathworks, MA) for two groups. We considered the group differences significant when *p* values were below 0.05.

## Results

### Evaluation of wrist movements in the three age groups

The movement kinematics for step-tracking wrist movements in the three age groups are shown in Fig 2. Fig 2A shows a typical example for the young group, Fig 2B for the middle-age group, and Fig 2C for the elderly group. In each panel, the flexion wrist movements to the left-sided target are shown in the left figure, while the extension wrist movements to the right-sided target are shown in the figure on the right side. Black lines in the inset for each example show the trajectories to reach the target from the onset of movement, while those in the top and bottom traces show angle X and velocity profiles. As shown in Fig 2, movement time was longer for the aged groups, while the maximum values of velocity profiles were lower for the aged groups regardless of the direction of movement. We first performed quantitative analysis of the wrist movements for the three groups in terms of the movement time and maximum velocity, as well as the reaction time.

#### 1) Age-related changes in reaction time (RT)

**Fig 3:**
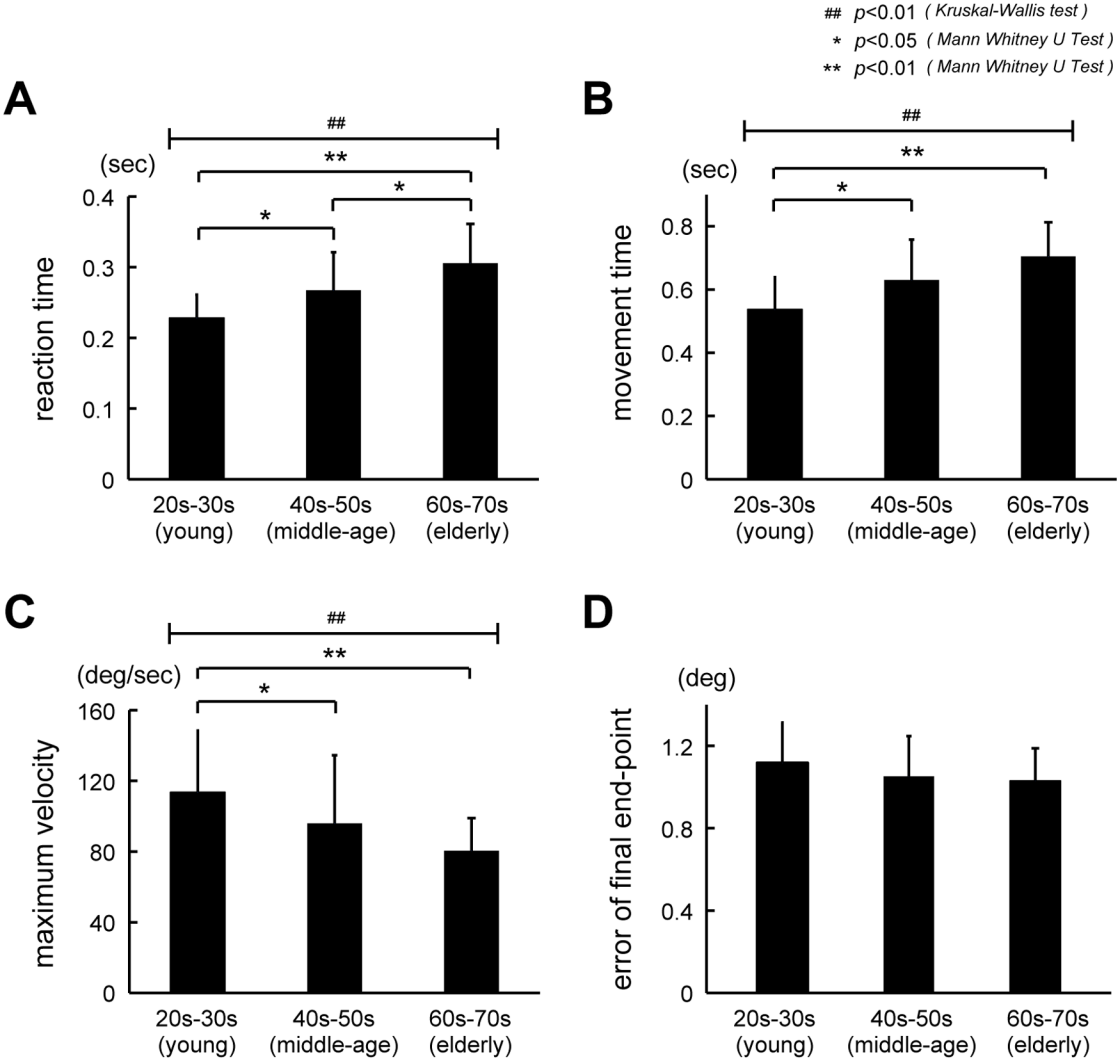
Age-related changes in kinematic properties for step-tracking wrist movements in terms of reaction time (RT), movement time (MT), maximum velocity (MVel), and terminal error. (A) Age-related differences in RT in the three age groups. There were statistically significant differences between all three groups (p< 1.5 × 10^−4^, Kruskal-Wallis test; p < 0.019 for young and middle-age; p < 0.03 for middle-age and elderly; p < 8.0 × 10^−5^ for young and elderly, Mann-Whitney U test). (B) Comparison of MT between the three age groups. There were statistically significant differences between the three age groups (p< 5.5 × 10^−4^, Kruskal-Wallis test), between the young group (20–30 years old) and the middle-age group (40–50 years old) (p < 0.033, Mann-Whitney U test), and between the young group (20–30 years old) and the elderly group (60–70 years old) (p < 1.25 × 10^−4^, Mann-Whitney U test). Note that the MT was longer for the aged groups. (C) Comparison of MVel between the three age groups. There were statistically significant differences among the three age groups (p< 0.003, Kruskal-Wallis test), between the young group (20–30 years old) and the middle-age group (40–50 years old) (p < 0.037, Mann-Whitney U test), and between the young group (20–30 years old) and the elderly group (60–70 years old) (p < 8.62 × 10^−4^, Mann-Whitney U test). Note that the MVel was lower for the aged groups. (D) Age-related changes in terminal error. The mean error values (± SD) for each age group were as follows: 1.12 deg (± 0.2) for the young group (n = 16), 1.05 deg (± 0.2) for the middle-age group (n = 16), and 1.03 deg (± 0.16) for the elderly group (n = 17). Note that there were no statistically significant differences among the three age groups (p<0.35, Kruskal-Wallis test).

The RT graphs of the three groups are shown in Fig 3A. The mean values (± SD) of RT for each age group were as follows: 0.23 s (± 0.03) for the young group (n = 16), 0.27 s (± 0.05) for the middle-age group (n = 16), and 0.31 s (± 0.06) for the elderly group (n = 17). RT was significantly longer for the higher age groups (p< 1.5 × 10^−4^, Kruskal-Wallis test; p < 0.019 for young and middle-age groups, p < 0.03 for middle-age and elderly groups, and p < 8.0 × 10^−5^ for young and elderly groups, Mann-Whitney U test).

#### 2) Age-related changes in movement time (MT)

The MT graphs of the three groups are shown in Fig 3B. The mean values (± SD) for each age group were as follows: 0.54 s (± 0.10) for the young group (n = 16), 0.63 s (± 0.13) for the middle-age group (n = 16), and 0.71 s (± 0.11) for the elderly group (n = 17). A comparison among the three groups showed a significant difference in MT (p< 5.5 × 10^−4^, Kruskal-Wallis test). In particular, MT in the young group was significantly lower than that in the middle-age and elderly groups (p < 0.033 for young and middle-age groups, p < 1.25 × 10^−4^ for young and elderly groups, Mann-Whitney U test). However, MT in the elderly group was not significantly different from that in the middle-age group (p < 0.101, Mann-Whitney U test). These results indicate that the middle-age and elderly groups took a significantly longer time than the young group to move the cursor to the target.

#### 3) Age-related changes in maximum velocity (MVel)

The MVel graphs of the three groups are shown in Fig 3C. The mean values (± SD) for each age group were as follows: 113.6 deg/s (± 34.7) for the young group (n = 16), 96.0 deg/s (± 38.5) for the middle-age group (n = 16), and 80.5 deg/s (± 18.5) for the elderly group (n = 17). As shown in Fig 3C, a comparison among the three groups demonstrated a significant difference in MVel (p< 0.003, Kruskal-Wallis test). MVel was significantly higher in the young group as compared to the middle-age and elderly groups f (p < 0.037 for young and middle-age, p < 8.62 × 10^−4^ for young and elderly, Mann-Whitney U test). However, no significant differences were observed between the middle-age and elderly groups (p < 0.257, Mann-Whitney U test). These result indicate that middle-age and elderly groups performed the step-tracking wrist movements with a lower peak velocity than the young group.

### Evaluation of control of preprogramed movement associated with aging

As shown in Fig 2A (A1 and A2), the red circles for the young group were concentrated near the target. However, in case of middle-age and elderly groups (Fig 2B and 2C), the variance of the red circles and their distance from the target had a strong tendency to be larger for the aged groups. Thus, we analyzed the difference in control ability of preprogramed movement between each age group in terms of the distance between the primary submovement and the target (i.e. error). We also examined age-related changes in the duration of the primary submovement.

#### 1) Parameter 1: Error of primary submovement

**Fig 4:**
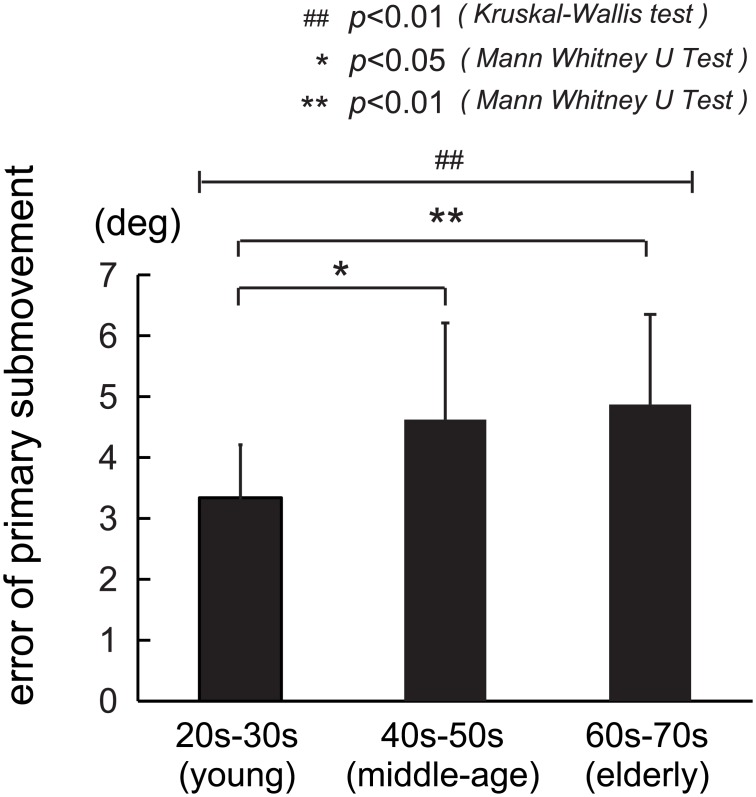
Age-related changes in errors following the primary submovement. The error of the primary submovement was measured as the distance between the starting point of the primary submovement and the target. Note that the error in the young group (20–30 years old) was significantly shorter than that in the other age groups, suggesting that the young group showed better performance in primary submovement than the middle-age and elderly groups, and that the accuracy of primary submovement was poorer for the aged groups.

We calculated the error of the primary submovement as the distance between the end (red circles in Fig 2) of the primary submovement and the center of the target, and compared the errors between the three groups in Fig 4. The mean values (± SD) for each age group were as follows: 3.34 deg (± 0.87) for the young group (n = 16), 4.62 deg (± 1.59) for the middle-age group (n = 16), and 4.87 deg (± 1.48) for the elderly group (n = 17). A comparison of the three groups showed a significant difference in the error (p< 0.004, Kruskal-Wallis test). The young group showed a significant difference as compared to the middle-age and elderly groups (p < 0.012 for young and middle-age groups, and p < 0.002 for middle-age and elderly groups, Mann-Whitney U test). However, the error in the elderly group was not significantly different from that in the middle-age group (p < 0.55, Mann-Whitney U test). These results suggest that the young group had better accuracy in primary submovement than the middle-age and elderly groups, as demonstrated by their cursors reaching closer to the target with the primary submovement (Fig 4).

#### 2) Parameter 2: Duration of primary submovement

**Fig 5:**
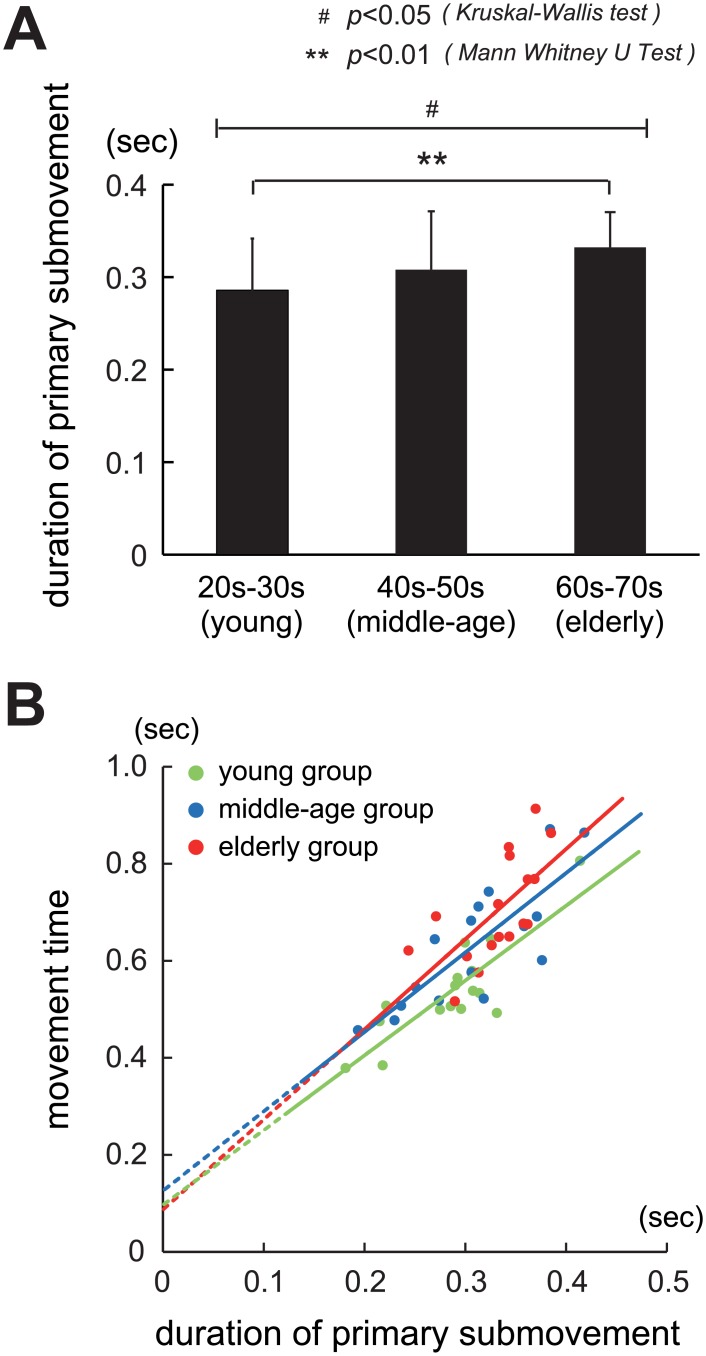
Age-related changes in the duration of primary submovement and its relationship with total movement time. (A) Comparison of the duration of the primary submovement in the three age groups. Note significantly longer duration of the submovement for the aged groups (p< 0.029, Kruskal-Wallis test). There was a statistically significant difference only between the young group (20–30 years old) and the elderly group (60–70 years old) (p < 0.004, Mann-Whitney U test). (B) Relationship between the duration of the primary submovement and movement time. Green, blue, and red points indicate the young group, the middle-age group and elderly group, respectively. Note that the ratio of the duration of the primary submovement to total movement time tended to be lower for the aged groups.

The durations of the primary submovement in the three age groups are shown in Fig 5. The mean values (± SD) for the duration of the primary submovement for each age group (Fig 5A) were as follows: 0.29 s (± 0.06) for the young group (n = 16), 0.31 s (± 0.06) for the middle-age group (n = 16), and 0.33 s (± 0.04) for the elderly group (n = 17). The duration of the primary submovement tended to be longer for the aged groups. While a significant difference was observed between the three groups (p< 0.029, Kruskal-Wallis test), two group comparisons revealed a significant difference only between the young and elderly groups (p < 0.004, Mann-Whitney U test).

Finally, we analyzed the relationship between the duration of the primary submovement and MT (Fig 5B), confirming the temporal properties of the primary submovement. As shown in Fig 5B, the duration of the primary submovement showed a strong correlation with MT, increasing as the speed of movement became slower. However, the correlation was lower for the higher age groups (R^2^ = 0.72 for the young group, 0.66 for the middle-age group, and 0.43 for the elderly group). Also, the ratio of the duration of the primary submovement to the total MT tended to be lower for the aged groups. This result suggests that the proportion of additional movements that require FB control for poorer control of preprogramed movement was higher for the aged groups.

## Discussion

In this paper, we examined age-related changes in control of preprogramed movement during the step-tracking movement of the wrist joint. We identified the primary submovement as the first bell-shaped velocity profile of the step-tracking movement, which is most likely under the FF control preprogrammed by the CNS [14–16]. We determined the end of the primary submovement based on the temporal profile of the tangential velocity [7] (Fig 2). Then, we examined the age-related changes in control of preprogramed movement as 1) the error (distance) between the end position of the primary submovement and the target, and 2) the duration of the primary submovement among in the three age groups. We found that young subjects demonstrated better performance in terms of the spatiotemporal property of primary submovement than middle-age and elderly subjects. In other words, the performance of primary submovement was lower for the aged groups (Figs 4 and 5A). We also found that the proportion of additional movements with FB control for poorer control of preprogramed movement was higher for the aged groups (Fig 5B). In the following discussion, we will focus on two points: 1) the primary submovement in step-tracking movements and FF control, and 2) age-related differences in motor control.

### Primary submovement in step-tracking movements and FF control

To date, several researchers have studied motor control using step-tracking movements [8, 9, 13] In general, it is most likely that FF control plays a primary role in the initiation of the step-tracking movement, while FB control supplements FF control near the end of the movement when required. Thus, if the subject moves a cursor into the target accurately with only FF control, they should show movements with a roughly straight trajectory and a bell-shaped tangential velocity profile [8, 9–12]. However, because FF control is not always perfect, they sometimes miss the target with the primary submovement, and therefore, need to generate additional movements with FB control (Fig 2).

Thus, in the present study, we assumed that the primary submovement, i.e. the first bell-shaped velocity profile represents the FF control preprogrammed by the CNS, while the second bell-shaped velocity profile represents the first FB control. We determined the end of the primary submovement by identifying the start of the second bell-shaped velocity profile. The start time of the first FB control (i.e. the end of the primary submovement,) was on average 320 ms for the young group, 350 ms for the middle-age group, and 360 ms for the elderly group, respectively (Fig 5A). The first FB control in the present study was later than reported in previous studies. For instance, Keele and Posner [29] reported that the timing to use vision to correct aiming movements on visual FB control is about 200 ms. Similarly, van Roon et al. [4] reported that the movement for any error correction based on visual error information requires 200 ms. In other words, the latter half of the primary submovement might be partly affected by FB control, therefore it might not be a pure FF control.

However, previous studies reported that the primary submovement in the goal-directed movement is most likely under the FF control preprogrammed by the CNS [7, 14–16]. In particular, Pratt and Abrams [15] reported that the subjects increase the distance and duration of primary submovements with practice, resulting in the increased percentage of the total movement traveled during the primary submovement. This means that the FF control is improved with practice. Accordingly, we instructed the subjects to maximize the primary submovement as the FF control with the following three instruction. First, as described in the methods section, we asked the subject to move the cursor as quickly and accurately as possible to the new target with “a single step” wrist movement. Therefore, the subjects were encouraged to maximize the primary submovement in the total movement as the FF control. Second, there is "the instruction period" in which both the initial target (the center target) and the secondary target (the new target) of the movement task are visible simultaneously (Fig 1C). This setup allowed the subjects to visually plan and execute the above mentioned strategy, where the subject maximized the primary submovement as the FF control. Finally, in order to make the subjects to understand the strategy of the movement task, the subjects performed a practice set of movements (8 movements) before collecting data. Consequently, these efforts prevented the subjects performing the movement task with strategically-segmented submovements or a smaller secondary movement.

Then we calculated the distance between the end position of the primary submovement and the target (i.e. error), in order to evaluate accuracy of FF control. Our results demonstrated that the young group showed better accuracy in FF control than the middle-age or elderly groups. Namely, the accuracy of FF control was poorer for the aged groups (Fig 4). Our results are similar to our previous studies in patients with cerebellar dysfunction. In other words, our previous studies showed that patients with cerebellar dysfunction demonstrated decline in the accuracy of movement and performed slower movements in the same step-tracking wrist movement task [30]. This demonstrates that the primary submovement and other parameters in the step-tracking movement can be used to evaluate the changes in motor control with cerebellar dysfunction, as well as age-related changes in motor control.

### Age-related differences and accuracy in motor control

In previous studies [1–3], some researchers quantified the motor function of elderly people by comparing the kinematic properties of the arm movements of an elderly group with those of a younger group. They reported the same results as in the present study, namely, longer RT and MT, and larger MVel and terminal error for the aged groups (Fig 3).

Age-related changes in FF control have been previously reported by van Roon et al. [4] in a study on motor development. They studied changes in the maximum velocity of hand movement towards a target in a manual tracking task for subjects ranging in age from 6 to 17 years. They reported increase in the maximum peak velocity with motor development. In particular, they reported the decrease in the number of velocity peaks, resulting in the smoother trajectories with development in children. They suggested that the development of motor control in children showed the change of control strategy from FB control to FF control. As expected from our results, our result demonstrated that the maximum peak velocity inversely correlated with the aging (Fig 3B). In the present study, we further looked into the overall temporal pattern rather than the momentary maximum value for the velocity alone (Fig 2). Specifically, we extracted some parameters that characterized the age-related changes in FF control from the primary submovement. Furthermore, we analyzed accuracy of the FF control by measuring the distance between the end points (red circles in Fig 2) of the primary submovement and the center of the target among the three age groups. Again, we found the lower accuracy of FF control for the aged groups (Fig 4). We also found the increase in duration for FF control, i.e. lower movement speed for the aged groups by measuring the duration of the primary submovement (Fig 5A). Although the duration of the primary submovement depends on movement speeds, a ratio of the duration of the primary submovement in the total movement time tended to be lower for the aged groups (Fig 5B). This result means that the proportion of additional movements with FB control to compensate the incomplete FF control was higher for the aged group.

Overall, the present study indicates that the primary submovement in the step-tracking movement provides useful parameters to evaluate the performance of FF control and its age-related changes.

### The scope of analysis in this study, and future directions

In this study, we analyzed the age-related changes in control of preprogramed movement for rapid aimed wrist movements. That is why there is no consensus among previous studies about the age-related changes in control of preprogramed movement for the rapid aimed movements, i.e. accuracy of primary submovement. In contrast, there is a consensus among previous studies about the age-related changes in FB control, suggesting that even elderly subjects preserve high reliability of FB control. However, for the control of preprogramed movement, some researchers reported significant age-related changes in the primary submovement [2,14,16,18–20], while others [3,17,19,21] reported that the changes were insignificant. In this study, we quantitatively examined the age-related changes in the spatio-temporal accuracy of the primary submovement in terms of our proposed parameters (i.e. the error and the duration of primary submovement)(Figs 4 and 5A). We found that, by comparing the errors between the three age groups (Fig 4), the spatial accuracy of primary submovement was poorer for the aged groups. For the temporal characteristics, we confirmed the slowness of the first movement in terms of the duration of primary submovement (Fig 5A). This result is consistent with that of previous studies [14,16], suggesting that these changes were not due to a specific strategy but to impairment in control of preprogramed movement for the aged groups.

In order to examine the age-related changes in the accuracy of primary submovement in the current digital era, it is important to consider the influence of experience of digital devices such as personal computers or smart phones. Indeed, we did not specifically design this study to consider the difference in experience of the digital devices for each age group. Nevertheless, as shown in Fig 2 and S1 Fig, the final end-point error was even smaller in the elderly group than the young and middle-age groups. In other words, the experience of digital devices was not a critical factor that altered the outcome of the present study. In contrast, there was a significant difference in terms of MT, MVel, and the error of primary submovement (Figs 3 and 4) between the young and middle-age groups, who were assumed to have comparable experience in digital devices. These changes should be age-related.

Finally, in this study, FF control and FB control were largely separated, because we employed a rapid-step tracking movement task. However, in our daily life, the two controllers work concurrently and therefore they are mostly inseparable. We need to find a new experimental paradigm to dissociate the two controllers in the more natural conditions. We are currently working on this issue.

## Supporting information

S1 Fig Age-related changes in error corrections by visual feedback (FB) including secondary movements. The mean values of error correction (± SD) for each age group were as follows: 2.22 deg (± 0.96) for the young group (n = 16), 3.57 deg (± 1.69) for the middle-age group (n = 16), and 3.84 deg (± 1.55) for the elderly group (n = 17).
(TIF)
