# Supplementary figures and images for: Quantitative evaluation of age-related decline in control of preprogramed movement
Source: PLoS One. 2017 Nov 29;12(11):e0188657. doi: 10.1371/journal.pone.0188657 (PMC5706693; doi:10.1371/journal.pone.0188657)

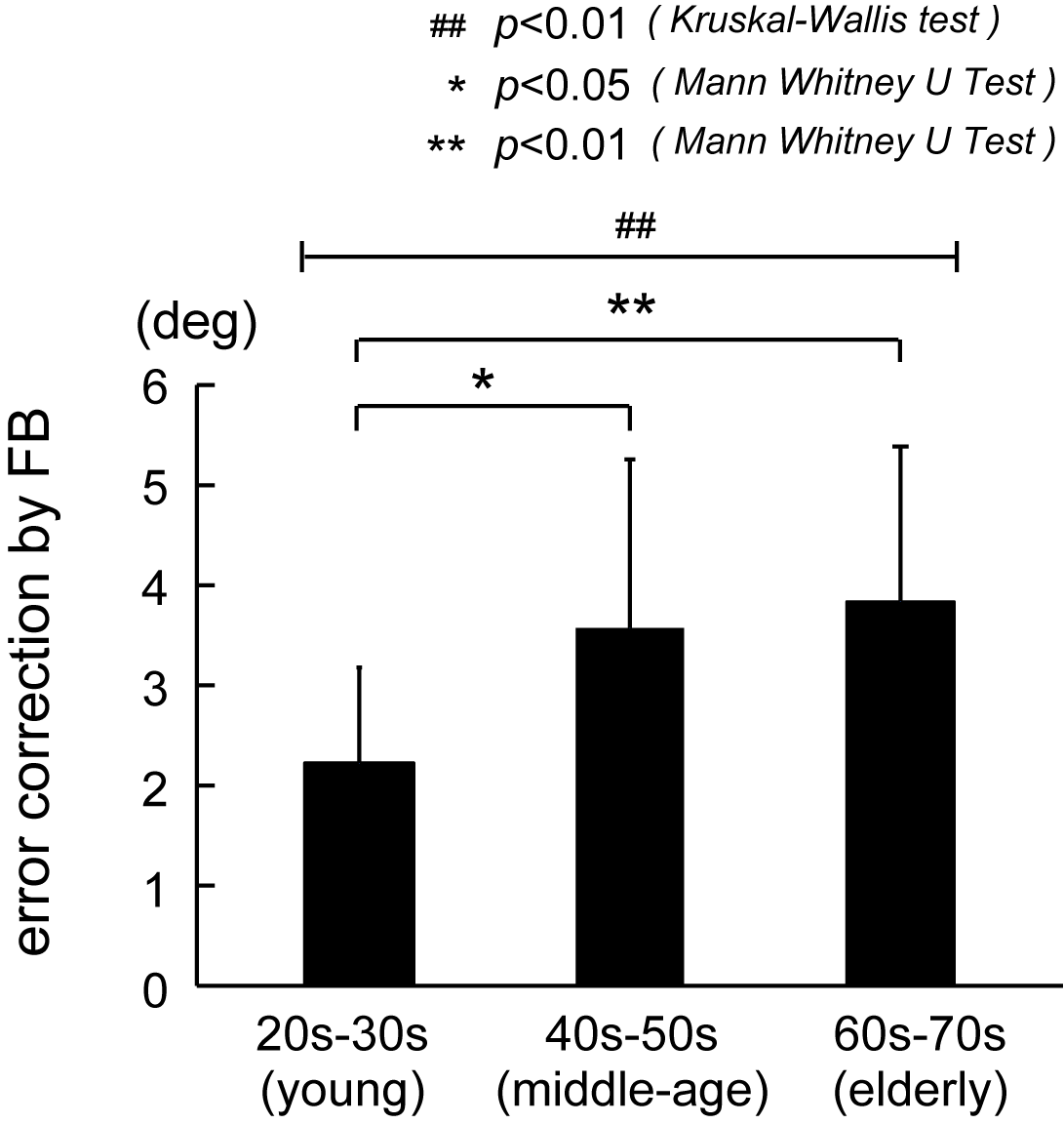

Supplement: S1 Fig — The mean values of error correction (± SD) for each age group were as follows: 2.22 deg (± 0.96) for the young group (n = 16), 3.57 deg (± 1.69) for the middle-age group (n = 16), and 3.84 deg (± 1.55) for the elderly group (n = 17). (TIF) [file pone.0188657.s001.tif]
